# 

**Published:** 1925

**Authors:** 


					CONTENTS. Page
The Diagnosis of Scarlet Fever.
By John Wallace, O.B.E.,
M.B., C.M.Ed.
157
The Comparative Efficiency of Local Anaesthetics.
By Eric
Watson-Williams, M.C., Ch.M., F.R.C.S.E,..;
164
Reviews of Books
172
Editorial Notes ...
188
Obituary :
Walter Carless Swayne,
M.D., B.S.Lond., M.D.,
Ch. B.Bristol ...
192
Eliza Walker Ddnbar,
M.D. Zurich, L.K.C.F.I., L.M.
197
The Library of the Bristol Medico-Chirurgical Society

				

